# Highly Efficient Piezoelectrets through Ultra-Soft Elastomeric Spacers

**DOI:** 10.3390/polym13213751

**Published:** 2021-10-29

**Authors:** Heinz von Seggern, Sergey Zhukov, Omar Ben Dali, Claas Hartmann, Gerhard M. Sessler, Mario Kupnik

**Affiliations:** 1 Department of Materials and Earth Sciences, Technical University of Darmstadt, Merckstr. 25, 64283 Darmstadt, Germany; 2 Department of Electrical Engineering and Information Technology, Technical University of Darmstadt, Merckstr. 25, 64283 Darmstadt, Germany; omar.ben_dali@tu-darmstadt.de (O.B.D.); claas.hartmann@tu-darmstadt.de (C.H.); g.sessler@nt.tu-darmstadt.de (G.M.S.); mario.kupnik@tu-darmstadt.de (M.K.)

**Keywords:** ferroelectret, piezoelectret, fluoropolymer, elastomer, FEP, TPU

## Abstract

Piezoelectrets are artificial ferroelectrics that are produced from non-polar air-filled porous polymers by symmetry breaking through high-voltage-induced Paschen breakdown in air. A new strategy for three-layer polymer sandwiches is introduced by separating the electrical from the mechanical response. A 3D-printed grid of periodically spaced thermoplastic polyurethane (TPU) spacers and air channels was sandwiched between two thin fluoroethylene propylene (FEP) films. After corona charging, the air-filled sections acted as electroactive elements, while the ultra-soft TPU sections determined the mechanical stiffness. Due to the ultra-soft TPU sections, very high quasi-static (22,000 pC N^−1^) and dynamic (7500 pC N^−1^) $d_{33}$ coefficients were achieved. The isothermal stability of the $d_{33}$ coefficients showed a strong dependence on poling temperature. Furthermore, the thermally stimulated discharge currents revealed well-known instability of positive charge carriers in FEP, thereby offering the possibility of stabilization by high-temperature poling. The dependences of the dynamic $d_{33}$ coefficient on seismic mass and acceleration showed high coefficients, even at accelerations approaching that of gravity. An advanced analytical model rationalizes the magnitude of the obtained quasi-static $d_{33}$ coefficients of the suggested structure indicating a potential for further optimization.

## 1. Introduction

Electrically charged microporous foams or voided polymer structures, commonly referred to as ferroelectrets or piezoelectrets [1,2], were introduced several decades ago and were reported to show a high longitudinal piezoelectric effect, the magnitude of which was much higher than the response of classical ferroelectric polymers such as PVDF [3,4] and even comparable to the best lead-based piezoceramics such as lead–zirconate–titanate (PZT) [5]. Along with other favorable characteristics, including mechanical flexibility, easy processability, good matching to water and, thus, also to the human body, as well as relatively low production costs, these materials represent a new type of electroactive polymers [6]. They have been considered for a wide range of applications in sensor and actuator technology [7,8,9] including energy harvesting gear for wearable electronics [10,11].

Depending on the pore structure, ferroelectrets can be divided into three groups: (a) ferroelectrets with a closed pore structure and lens-like air voids [9,12,13,14,15,16], (b) multilayer configurations with open-porous foams embedded between two thin, solid polymer films [17,18,19,20,21,22,23], and (c) polymer arrays with artificially introduced air voids [24,25,26,27,28,29,30,31]. All of the above structures are originally non-polar and must be polarized in a sufficiently large external electric field to exhibit piezoelectricity. During the poling process, an electrical breakdown in air (i.e., Paschen breakdown [32]) occurs in the air-filled voids thereby creating free charges of opposite sign. These charges are separated by the applied electric field and subsequently move to the opposite interfaces of the air voids or to the solid polymer layers. There, the charges are quasi-permanently trapped. The resulting polarization $\sigma_{\mathrm{rem}}$ along the void interfaces constitute an important factor for the piezoelectricity of ferroelectrets [1,2].

**Figure 1:**
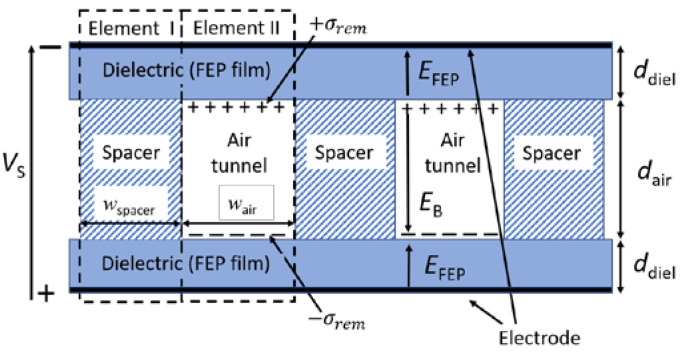
Schematic cross-section of a generalized structure of a ferroelectret.

Despite the large variety of possible porous structures, the structure of a particular ferroelectret, shown in Figure 1, can exemplarily be used to analyze the poling behavior and the electromechanical response of even more complicated structures. The sandwich in Figure 1 can be described by two thin layers of a polymer dielectric, in the present case of Teflon FEP-A, alternatively separated by an air tunnel and a spacer. Therefore, the structural unit of the sandwich consists of two basic elements which are represented by dielectric/spacer/dielectric (Element I) and dielectric/air tunnel/dielectric (Element II) arrangements.

All structures with air voids exhibit a limit for the remanent polarization $\sigma_{\mathrm{rem}}^{\max}$, which depends on the electric breakdown field $E_{B}$, the air gap thickness $d_{\mathrm{air}}$, the thickness of the polymer dielectric $d_{\mathrm{diel}}$, and their dielectric constants $\varepsilon_{\mathrm{air}}$ and $\varepsilon_{\mathrm{diel}}$. For the sandwich shown in Figure 1, this limit is [33]:

(1) $$\sigma_{\mathrm{rem}}^{\max}=\alpha \cdot \left(\varepsilon_{\mathrm{air}}\varepsilon_{0}+\varepsilon_{\mathrm{diel}}\varepsilon_{0}\frac{d_{\mathrm{air}}}{2d_{\mathrm{diel}}}\right)\;E_{B\;},\;$$

where $\alpha =\frac{w_{\mathrm{air}\;}}{w_{\mathrm{air}}+w_{\mathrm{spacer}}}$ defines the ratio of the area covered by the air tunnel to the total area, and $w_{\mathrm{air}\;}$ and $w_{\mathrm{spacer}\;}$are the width of the tunnel and spacer, respectively. Thus, the total length and thickness of both elements is equal, guaranteeing that the area and volume of Elements I and II are proportional to its respective width. The ratio α must be taken into account, since the polarization generated in Element II under mechanical stress must be averaged over the entire electrode area of the sample, whereas Element I contributes only partially or not at all to the total polarization [22,34]. It should be noted that Equation (1) only holds for a strictly plane-parallel configuration of air tunnels and dielectric films. For an air cavity in the form of a lens or a spherical cap, it is necessary to make corrections as discussed elsewhere (e.g., [34,35,36]). In addition to the theoretical model for the polarization, the maximum longitudinal piezoelectric $d_{33}^{\max}$ coefficient for the sandwich, shown in Figure 1, can be expressed as [21,37]:

(2) $$d_{33}^{\max}=\frac{\varepsilon_{\mathrm{diel}}\varepsilon_{\mathrm{air}}\;\sigma_{\mathrm{rem}}^{\max}}{Y_{\mathrm{total}}}\;\cdot \;\frac{1+\left(d_{\mathrm{air}}/2d_{\mathrm{diel}}\right)\;}{{\left(\varepsilon_{\mathrm{air}}+\varepsilon_{\mathrm{diel}}\left(d_{\mathrm{air}}/2d_{\mathrm{diel}}\right)\right)}^{2}}\;,\;$$

where $Y_{\mathrm{total}}$ represents the Young’s modulus of the entire structure, mainly determined by the stiffness of the spacers acting as a restoring force in order to maintain the air gap (Figure 1). Note that Equation (2) is based on an analytical model originally proposed for cellular structures [38,39]. According to Equation (2), the accumulated polarization $\sigma_{\mathrm{rem}}^{\max}$ at the air/polymer interface and Young’s modulus $Y_{\mathrm{total}}$ are the two key parameters that determine the final magnitude of the piezoelectric response. Both parameters must be optimized to obtain the maximum piezoelectric performance as discussed in several recent reviews [9,10].

In this work, a different strategy was used to increase the overall piezoelectric response. Ultrasoft thermoplastic polyurethane (TPU) [40,41] with a very low Young’s modulus was used as spacer material in order to significantly reduce the apparent stiffness of the device, while dielectric films of polyfluoroethylene propylene (FEP) were chosen as dielectric films to keep the polarization characteristics of the air tunnels similar to previous publications [18,19,20,21,22,23,24,25,26,27,28,30,31,33,34,37]. The question of how geometrical, electrical, and mechanical parameters influence the $d_{33}$ piezocoefficient was the main objective of the present publication. In addition, issues related to the optimal charging conditions and the stability of the piezoelectric coefficients over time and their dependence on mechanical stress and frequency were experimentally investigated and are discussed.

## 2. Materials and Methods

**Figure 2:**
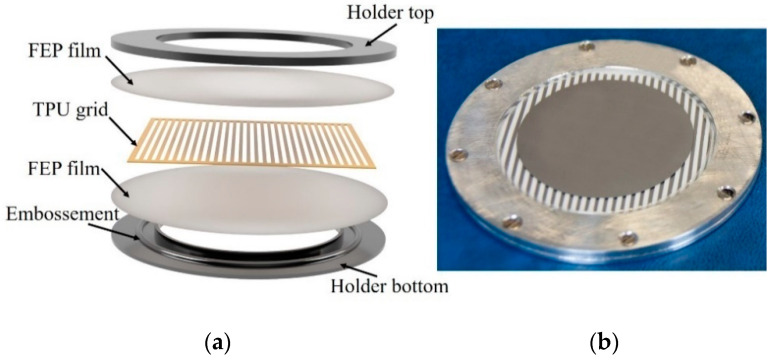
(**a**) Sandwich structure consisting of a 3D-printed Varioshore TPU frame with FEP layers on the top and bottom. All three layers were clamped together using an aluminum holder with tensioning groves as described in the text. (**b**) Photograph of the FEP/TPU-air/FEP device equipped with Al electrodes.

The polymer sandwiches were fabricated from two 25 µm thick polyfluoroethylene propylene films (FEP-A, Sheldahl Ltd., Northfield, MN, USA) embedding a grid of thermoplastic polyurethane (VARIOSHORE TPU NATURAL, colorFabb B.V., DK Belfeld, The Netherlands) as depicted in Figure 2a. The TPU grid was printed on a Prusa MK3s 3D printer (Prusa Research, Prague, Czech Republic). The 3D structure was outlined using the software package Autodesk Fusion 360 (Autodesk Inc., San Rafael, CA, USA) and sliced using the open-source software SuperSlicer 2.3.56 based on PrusaSlicer (Prusa Research, Prague, Czech Republic). The slicing software helped to convert the 3D structure into layers with defined print parameters, such as layer thickness, printing velocity, and extruder temperature, which are listed in Table 1.

All printed TPU spacers were 270 ± 5 µm thick with dimensions 50 × 50 mm^2^ and consisted of alternating TPU strips and air tunnels (Figure 2a). The width of the air tunnel $w_{\mathrm{air}}$ was initially equal to the width of the TPU stripe $w_{\mathrm{TPU}}\;$and amounted to 1 mm. Later, the TPU stripes were cut out to obtain 3 mm wide air tunnels.

The sandwich of alternating FEP/TPU/FEP and FEP/air/FEP elements was clamped in an aluminum holder with an inner diameter of 45 mm and served as support for electrical and mechanical testing. An additional ring-shaped embossment on the bottom part and a matching groove on the top part of the holder allowed for the maintenance of equilateral pre-stress on the sandwich. During preparation, any excess air in the sandwich was eliminated by an initial evacuation. Afterwards, the sandwich was perforated by punching small holes in the non-metallized outer periphery of each air tunnel outside the measuring or poling area to allow the air channels to return to normal air pressure. The total thickness of the prepared sandwiches was 320 ± 5 μm. Depending on the performed experiment, one or both sides of the sample were metallized with 100 nm thick circular aluminum electrodes (Ø 42 mm) by thermal vapor deposition on the rear side of the bottom FEP layer or top and rear sides of the FEP layers.

The samples were polarized by a corona triode in air under ambient conditions (atmospheric pressure and a relative humidity of 50%) utilizing a DC corona voltage of $V_{C}\;$= −10.0 kV and a DC grid voltage $V_{G}$ between 0 and −4.0 kV to achieve the desired surface potentials. The samples were either charged at temperatures of 25 or 120 °C. The poling experiment was carried out with one-sided metallized sandwiches with the non-metallized surface facing the corona grid. Three signals were recorded simultaneously during poling: the DC charging current, $I_{C}$; the surface potential, $V_{S}$; the photomultiplier current, *I*_PM_. The charging current was measured by means of an electrometer (Keithley 600B) and the surface potential, $V_{S}$, by the Kelvin technique. A photomultiplier (Hamamatsu, model R6094) was used to monitor the light emission during poling initiated by Paschen breakdown in the air tunnels [42].

In order to experimentally determine the quasi-static piezoelectric $d_{33}$ coefficient, an aluminum electrode 42 mm in diameter was vacuum-deposited onto the non-metallized top surface of the poled sample followed by short-circuiting. The coefficient was determined by applying a defined weight of mass *m* to the device in short-circuit prior to measurement. Afterwards, the mass was quickly removed, and the generated charge Q was recorded for 10 s using a Keithley 6517 electrometer. The $d_{33}$ coefficient then followed as:

(3) $$d_{33}=\frac{Q}{mg}=\frac{\sigma_{0}}{\sigma_{\mathrm{mech}}}\;,$$

where *g* is the acceleration of gravity, $\sigma_{0}=Q/A$ is the induced surface charge density, and $\sigma_{\mathrm{mech}}=mg/A$ is the stress released from the sample of electrode area *A*.

In order to measure the dynamic $d_{33}$ coefficient, a seismic mass, $m_{S}$, was placed on top of the sample which was mechanically excited by an electrodynamic shaker table (B&K 4809). By measuring the generated charge and the dynamic force, the dynamic $d_{33}\;$coefficient as a function of frequency could be determined by the audio analyzer *d*Scope Series III (Prism Sound Ltd., Ely, UK) and a buffer power amplifier (B&K 2706). More information on the experimental setup is published elsewhere [43].

In order to understand the thermal charge stability, samples were polarized at different temperatures and the thermally stimulated depolarization currents were measured in open-circuit [44,45,46]. Therefore, a gold-plated metal electrode was placed 4 mm above the non-metallized surface of the corona-poled sample, and the discharge currents were monitored by a Keithley electrometer (model 610C) utilizing a constant heating rate of 3 K min^−1^.

Stress–strain curves under compression were taken at room temperature by a universal testing machine (Inspekt table 5 kN, Hegewald & Peschke) in order to investigate the mechanical properties of the 3D-printed TPU films. Thus, the specimens were compressed between two parallel plates at a rate of 0.1 N s^−1^, and the resultant force was recorded by a force sensor (100 N, HBM) allowing for the determination of the Young’s modulus in the thickness direction.

## 3. Results and Discussion

### 3.1. Poling of Elastomer-Spaced Fluoropolymer Piezoelectrets

In order to obtain devices with maximum piezoelectric coefficients, an optimal poling voltage must be used. According to theoretical models for similar devices with two solid blocking layers separated by an air gap [21,33], an increase in the poling voltage, *V*, across the entire structure results in an increase in the electric field in the air gap,$\;E_{\mathrm{air}}$, and across the FEP films, $E_{\mathrm{FEP}}$. When $E_{\mathrm{air}}$ reaches the breakdown field strength of air, $E_{B}$, breakdown starts in the air tunnels. This occurs at the surface potential $V_{B}$ of the sandwich, also denoted as the breakdown voltage. It yields [33]:

(4) $$V_{B}=\left(d_{\mathrm{air}}+\frac{2\varepsilon_{\mathrm{air}}d_{\mathrm{FEP}}}{\varepsilon_{\mathrm{FEP}}}\right)\;E_{B}\;,$$

where $d_{\mathrm{FEP}}\;$and $d_{\mathrm{air}}$ denote the thickness of the FEP films and the air tunnels with relative dielectric permittivity $\varepsilon_{\mathrm{FEP}}$ and $\varepsilon_{\mathrm{air}}$, respectively. All parameters on the right side of Equation (4) are experimentally accessible except for $E_{B}$, which can be taken in principle from the well-known Paschen law for air [32]. Since, however, in a number of cases the experimentally determined value for this parameter significantly differed from the values predicted by Paschen’s law [33,47], it seems advisable to determine this parameter specifically for the here utilized sandwiches. This can be done by measuring the breakdown voltage, $V_{B}$, which on the one hand determines $E_{B}$ by means of Equation (4) and on the other hand, by means of Equation (1), the maximum residual polarization, $\sigma_{\mathrm{rem}}^{\max}$ [33]. It should be noted, as shown in Equation (1), that $\sigma_{\mathrm{rem}}^{\max}$ must be corrected by the ratio of the polarized to the total sample area when only a part of the sample is polarized.

**Figure 3:**
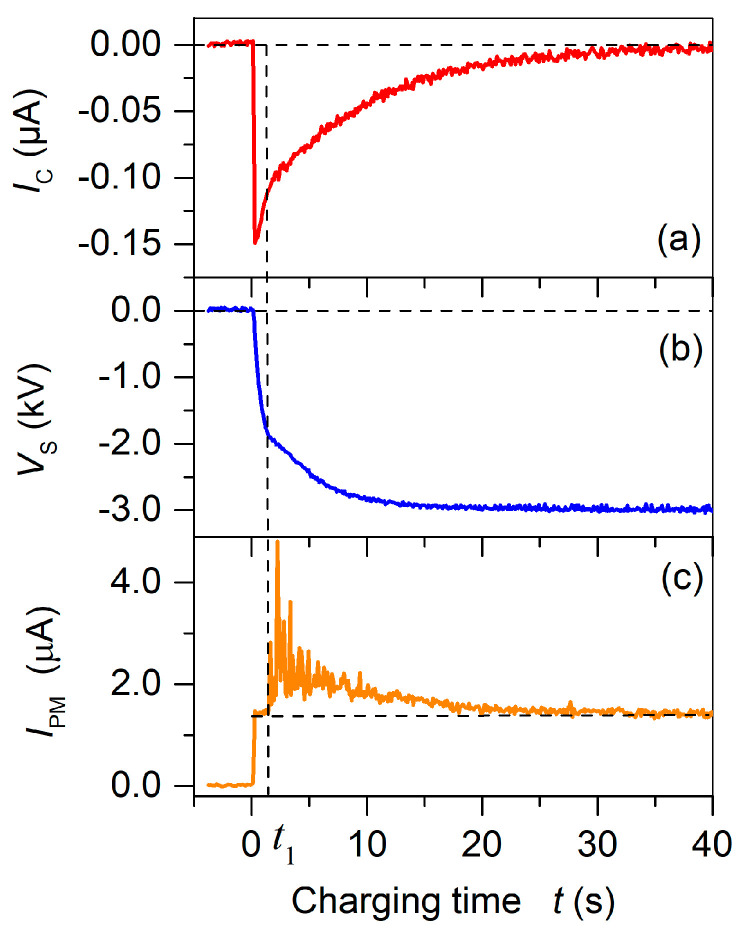
Temporal dependencies of (**a**) charging current $I_{C}$, (**b**) surface potential $V_{S}$, and (**c**) photomultiplier current $I_{\mathrm{PM}}$ for poling of a virgin sandwich of alternating FEP/TPU/FEP and FEP/air/FEP elements with a width ratio of 1 at $V_{G}\;$ = −3.0 kV. The corona voltage was switched on at time t = 0 s. Time $t_{1}$ indicates the onset of breakdown-induced light emission and polarization of the sample.

Instead of measuring the hysteresis for the experimental determination of $V_{B}$ [1,22,23,37], the light emission at breakdown onset was used [6,42]. Figure 3 compares the temporal evolution of the poling current, the surface voltage built up, and the photomultiplier current, $I_{\mathrm{PM}}$, for the poling process of a virgin one-sided metallized sandwich. For a grid voltage of −3.0 kV, the poling process starts at t = 0 s when the corona and grid voltages are switched on. As soon as the corona voltage is applied, light is emitted from the plasma discharge at the tip of the corona needle which remains constant during the complete charging process at a level indicated by the vertical dashed line in Figure 3c. This light emission is, however, not related to the air-breakdown, leading to polarization of the sample. The actual onset of the breakdown-induced polarization is seen by the increase in the light intensity starting at $t=t_{1}$. At this moment, the surface potential has reached the breakdown voltage $V_{B}\;$= −1.9 kV. The change in the slopes of the surface potential (Figure 3b) and the charging current (Figure 3a) indicate the onset of the poling process. The build-up of the polarization is terminated after approximately 35–40 s. At this time, the poling current $I_{C}$ and the light intensity $I_{\mathrm{PM}}$ both approach values of zero, while the surface potential saturates at a value close to the applied grid potential $V_{G}$. Utilizing the so determined $V_{B}$ and the experimental values of $d_{\mathrm{FEP}}$ = 25 µm, $d_{\mathrm{air}}$ = 270 µm, $\varepsilon_{\mathrm{FEP}}$ = 2.1, and$\;\varepsilon_{\mathrm{air}}\;$= 1, Equation (4) delivers $E_{B}$ = 65.0 kV cm^−1^, which is in excellent agreement with the value predicted by Paschen’s law of 66.1 kV cm^−1^. Since theory predicts that the maximum remanent polarization, $\sigma_{\mathrm{rem}}^{\max}$, for such a geometry is reached at $V_{S}=2\times V_{B}$ [33], the utilized poling voltage of 4.0 kV is sufficient to obtain the maximal possible polarization for the present FEP/TPU/FEP–FEP/air/FEP sandwiches with a thickness of 320 ± 5 µm.

### 3.2. Temporal and Thermal Stability of the d_33_ Coefficient

**Figure 4:**
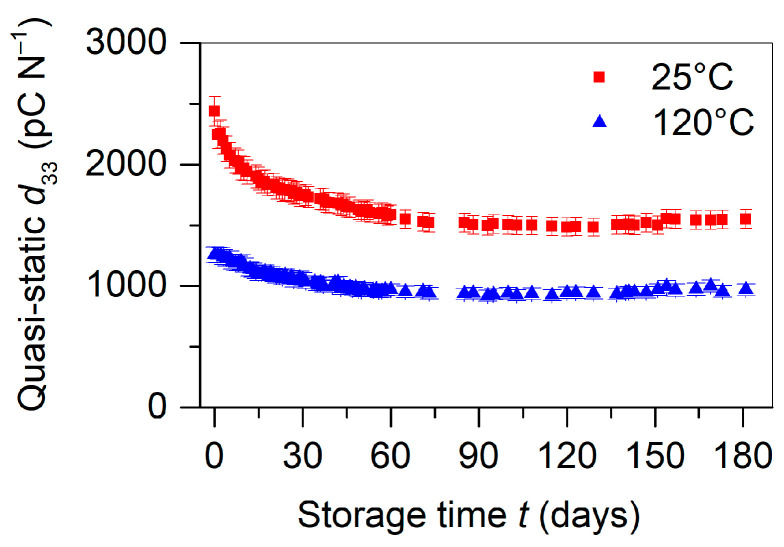
Isothermal decay of an averaged piezoelectric $d_{33}$ coefficient for five similar alternating FEP/TPU/FEP and FEP/air/FEP samples, all with a width ratio of 1, polarized at 25 and 120 °C, respectively, and measured at room temperature. Measurements of the $d_{33}$ coefficients were performed at a static load of 1.2 kPa.

A first important property of the fabricated structure is its temporal stability of piezoelectricity under ambient conditions. After maximal polarization of a batch of five equally prepared samples at room temperature (25 °C), the mean piezoelectric $d_{33}$ coefficient was measured periodically over six months under ambient conditions using a static stress of 1.2 kPa (see Figure 4). It showed a high initial piezoelectric response of approximately 2430 pC N^−1^ and a decrease over the next 180 days, indicating an average loss of 40% of the initial piezoelectricity. The temporal loss of the $d_{33}$ coefficient can be attributed to the poor charge storage stability of the positive charge in FEP [46]. Since during poling an equal amount of positive and negative charge is generated and trapped at the FEP/air interfaces of the air-filled tunnels, the temporal stability of the positive charge will dominate the overall discharge process. The instability of the positive charges explains the order of magnitude of the observed loss of approximately 40% for the $d_{33}$ coefficient, since it is known from the literature that a small fraction of the positive charge can also be deeply trapped [46]. On the other hand, the charge storage properties of TPU have not been well investigated so far. Preliminary experiments indicate, however, a high thermal conductivity, since all attempts to charge individual TPU films by corona triode were unsuccessful. Therefore, we considered the FEP/TPU/FEP element as piezoelectrically inactive due to the quick discharge of possible poling-induced charges on the FEP layers in these elements.

It is, however, also known from the literature that a positive charge in FEP can be stabilized by poling FEP films [46] and also fluoropolymer-based ferroelectrets [20,24,31,34] at elevated temperatures. The physics behind this stabilization is explained by the existence of a large amount of shallow hole traps and a relatively small amount of deep hole traps [46]. Poling at elevated temperatures allows the shallow traps to empty spontaneously and for the charge to transit the sample quickly. This transit is accompanied by a gradual filling of the deep traps which prevail at the end of poling. In order to determine how this approach is applicable to the systems studied in this research, a batch of manufactured devices was polarized at 120 °C, and the isothermal decay of the $d_{33}$ coefficients was investigated. The corresponding experimental results for the averaged piezoelectric $d_{33}$ coefficient for five samples are shown in Figure 4. The strongly reduced decay observed for the 120 °C poled structure can be explained by the improved stability of the positively charged FEP when charged at an elevated temperature [46]. The still observed decay for the high-temperature poled samples is not clearly understood, but it may be related to charging of the residual air gaps near the FEP/TPU interfaces, the physics of which has yet to be explored in detail in upcoming studies.

**Figure 5:**
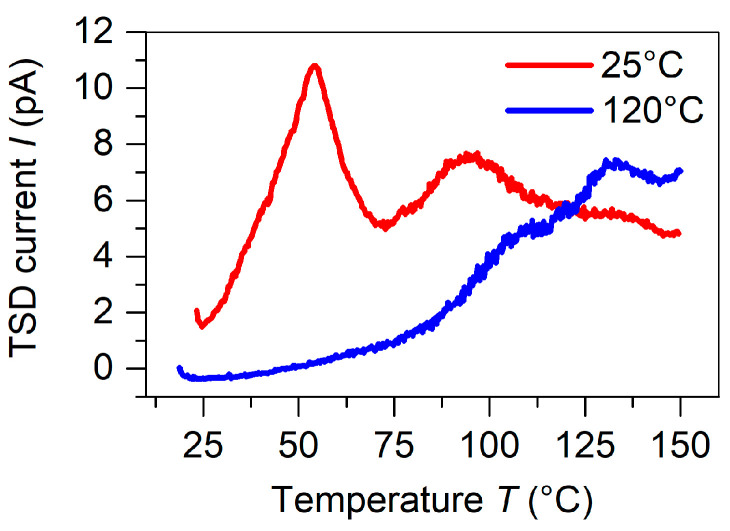
Open-circuit TSDC spectra measured for alternating FEP/TPU/FEP and FEP/air/FEP devices with a width ratio of 1 charged in a corona triode utilizing $V_{C}\;$= −10.0 kV and $V_{G}\;$ = −4.0 kV and two different temperatures as indicated.

Thermally stimulated depolarization current (TSDC) experiments were carried out for both charging temperatures under open-circuit conditions to understand more thoroughly the reasons for the increase in thermal stability of the sandwich charged at elevated temperatures. Figure 5 compares the TSDC spectra for samples corona charged at 25 and 120 °C using a grid potential of −4.0 kV and a poling time of five minutes resulting in a surface potential of $V_{S}\;$= −3.9 kV and −3.0 kV, respectively. The temperature of 120 °C was chosen as the maximum poling temperature to avoid thermal degradation and deformation of the TPU elastomer. At the same time, the TSDC measurements had to be limited to a maximum temperature of 150 °C, the highest temperature possible before destroying the samples. Three peaks at 50, 95, and 130 °C can be well distinguished in the samples polarized at 25 °C, while only two overlapping peaks presumably at 95 and 130 °C remained in the sample poled at 120 °C. The first peak at 50 °C completely disappeared for the sample poled at 120 °C, the second peak at 95 °C decreased significantly, and the peak at 130 °C increased.

For the assignment of the polarity of the trapped charge carriers to the respective detrapping peaks of the TSDC spectra in Figure 5, it is known from the literature that holes release shallow traps mainly at 50 °C and deep traps at temperatures distributed in the range from 90 to 200 °C [46,48], while electron detrapping occurs between 120 and 150 °C as well as between 170 and 220 °C [44,45,49]. The observed discharge currents can then be explained as follows: For the poling at room temperature, holes filled preferentially shallow traps indicated by the pronounced TSDC peak at 50 °C. The other two peaks were correlated to deeper hole traps (at 95 °C) and to electrons traps (at 130 °C). For the charging at 120 °C, the shallow traps at 50 °C were no longer trapping holes, since charges were released immediately after capture. Thereafter, the deeper hole traps filled gradually, dependent on their trap density. Contrary to the negative trap density, the trap density for deep holes was limited in FEP [46]. As a result, the total amount of captured charge would be less in a device polarized at an elevated temperature and the surface potential would also be less than that possible by the grid potential of the corona poling setup. In practice, the final surface potential for the sample polarized at 120 °C was $V_{0}\;$= −3.0 kV for a grid voltage of −4.0 kV. The consequence was that the sample polarized at 120 °C possessed a smaller piezoelectric response, whereas its stability over time and temperature was significantly improved, which can be seen in Figure 4. The sample charged at 120 °C exhibited approximately half the value of the initial piezoelectric $d_{33}$ coefficient compared to the device polarized at 25 °C, but only lost approximately 20% of its initial value after 180 days of storage under laboratory conditions, whereas the room temperature charged device lost approximately 40% of its initial value. The reason for the 20% remains unknown.

### 3.3. Mechanical Properties and Dependence of the d_33_ Coefficient on Pressure, Frequency, and Acceleration

According to Equation (2), besides the remanent polarization $\sigma_{\mathrm{rem}}^{\max}$, the effective Young’s modulus in the thickness direction $Y_{\mathrm{total}}$ of the complete sandwich plays a decisive role in the achievable piezoelectric $d_{33}$ coefficient [21,37,38,39]. It was obvious that the effective mechanical modulus of the alternating FEP/TPU/FEP–FEP/air/FEP sandwich was mainly determined by the mechanical properties of the TPU layer. It is known from the literature that FEP has a Young’s modulus ($Y_{\mathrm{FEP}}$) of approximately 500 MPa [50,51], while the modulus of TPU elastomers ($Y_{\mathrm{TPU}}$) is usually approximately 50 times smaller [41,52]. In addition, it may vary greatly depending on the 3D print parameters [41]. Therefore, in order to predict the mechanical properties of the alternating FEP/TPU/FEP–FEP/air/FEP sandwiches, it is necessary to determine the mechanical properties of the printed TPU elastomer used in this work.

**Figure 6:**
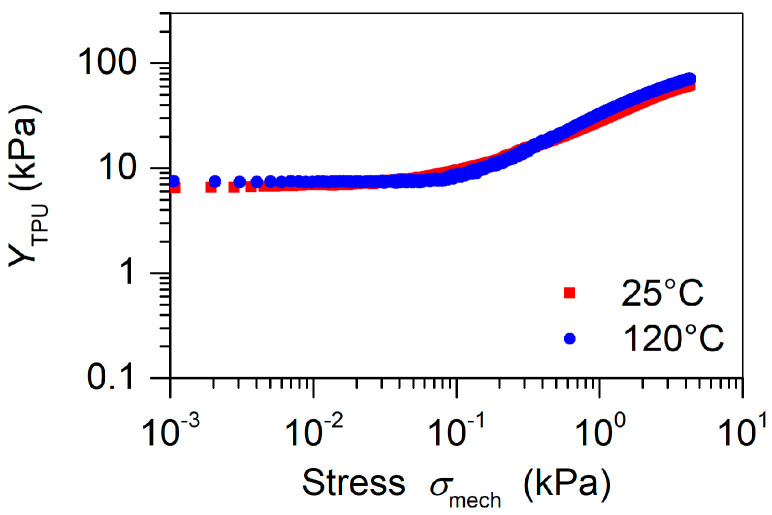
$Y_{\mathrm{TPU}}$ versus compression stress for non-annealed (25 °C) and annealed (at 120 °C) solid TPU films of 270 µm thickness.

To this end, bulk TPU films (i.e., films without air tunnels) with a thickness of 270 ± 5 µm were fabricated with the 3D printing technique utilizing the parameters from Table 1. Several films were annealed at 120 °C for 30 min to check how the thermal treatment during corona poling affects the mechanical properties of the TPU elastomer. Then, quasi-static stress–strain dependencies were measured at room temperature for non-annealed and annealed films for strain levels $\varepsilon_{\mathrm{mech}}$ up to 0.1. It turned out that bulk TPU films treated at the two temperatures exhibited very similar mechanical responses. Figure 6 compares the Young’s moduli $Y_{\mathrm{TPU}}=\partial \sigma_{\mathrm{mech}}/\partial \varepsilon_{\mathrm{mech}}$ for non-annealed and annealed films at different stress levels. It can be observed that for 3D-printed films, the Young’s moduli at low stress (˂0.1 kPa) were constant at approximately 7–8 kPa for both films, which is a factor 1000 times smaller than the published values for solid TPU [41,52]. Such low values indicate an open porous, air-filled structure of the printed TPU, and the constant Young’s moduli for stress up to 0.1 kPa indicates that the TPU follows Hooke’s law. At higher stress levels, the stiffness of both films increases to approximately 50 kPa, which suggests a densification of the open porous TPU polymer. Taking these extremely low Young’s moduli for TPU, the stiffness of the alternating FEP/TPU/FEP–FEP/air/FEP sandwiches was mainly determined by the intrinsic mechanical properties of the TPU elastomer for open air tunnels and was practically independent of the thermal pretreatment of the TPU up to 120 °C.

Since the two elements of the alternating FEP/TPU/FEP–FEP/air/FEP sandwich were clearly separated into electrical and mechanical properties, one can use the classic Voigt and Reuss approaches to model the Young’s moduli of the complete sandwich as shown in Figure 1 [53,54]. Therefore, Elements I and II of the manufactured sandwich can be considered as two parallel elements. Element I consisted of a three-layer structure of FEP/TPU/FEP with the width $w_{\mathrm{TPU}}$, and Element II consisted of a three-layer structure FEP/air/FEP with the width $w_{\mathrm{air}}$, where the lengths and thicknesses of those elements were the same as mentioned previously. For such parallel arrangements, the Voigt model can be used to calculate the total Young’s modulus of the combination of Elements I and II as follows:

(5) $$Y_{\mathrm{total}}=f\;Y_{E_{1}}+\left(1-f\right)\;Y_{E_{2}}\;,$$

where *f* is the volume fraction of Element I, and $Y_{E_{1}}$ and $Y_{E_{2}}$ are the Young’s moduli of Elements I and II, respectively. Since, as already mentioned above, the TPU stripes and air tunnels are of the same total length and height, *f* can be reduced to the width of the TPU stripes and air tunnels $w_{\mathrm{TPU}}$ and$\;w_{\mathrm{air}}$, respectively, and can be written as:

(6) $$f=\frac{w_{\mathrm{TPU}}}{w_{\mathrm{TPU}}+w_{\mathrm{air}}}\;\mathrm{and}\;1-f=\frac{w_{\mathrm{air}}}{w_{\mathrm{TPU}}+w_{\mathrm{air}}}\;.$$

Then, the Reuss model was adopted to calculate the Young’s moduli of the elements $E_{1}$ and $E_{2}$. It yields under the assumption of constant stress throughout each element:

(7) $$Y_{E_{1}}=\frac{2d_{\mathrm{FEP}}+d_{\mathrm{TPU}}}{\frac{2d_{\mathrm{FEP}}}{Y_{\mathrm{FEP}}}+\frac{d_{\mathrm{TPU}}}{Y_{\mathrm{TPU}}}}\;\mathrm{and}\;Y_{E_{2}}=\frac{2d_{\mathrm{FEP}}+d_{\mathrm{air}}}{\frac{2d_{\mathrm{FEP}}}{Y_{\mathrm{FEP}}}+\frac{d_{\mathrm{air}}}{Y_{\mathrm{air}}}}\;,$$

where $Y_{\mathrm{air}}$ is the Young’s modulus of air, which for non-sealed air tunnels can be assumed to be equal to zero. Taking additionally into account that $Y_{\mathrm{FEP}}\;\gg \;Y_{\mathrm{TPU}}$, these two simplifications combined with Equations (5)–(7) yield:

(8) $$Y_{\mathrm{total}}=\frac{w_{\mathrm{TPU}}}{w_{\mathrm{TPU}}+w_{\mathrm{air}}}\times \frac{\;\left(2d_{\mathrm{FEP}}+d_{\mathrm{TPU}}\right)}{d_{\mathrm{TPU}}}\;\cdot Y_{\mathrm{TPU}}\;.$$

Introducing Equations (1) and (8) into Equation (2), one obtains for the maximal obtainable $d_{33}^{\max}$ coefficient:

(9) $$d_{33}^{\max}=\frac{w_{\mathrm{air}}}{w_{\mathrm{TPU}}}\times \frac{\varepsilon_{0}\;\varepsilon_{\mathrm{FEP}}\;E_{B}\;}{Y_{\mathrm{TPU}}\;\left(\frac{2d_{\mathrm{FEP}}}{d_{\mathrm{TPU}}}+\varepsilon_{\mathrm{FEP}}\right)}\;.$$

At this point it should be emphasized that Equation (9) can be used as a guide for the further optimization of elastomer-spaced piezoelectrets. It can easily be seen that one possibility is to diminish the denominator $\left(\frac{2d_{\mathrm{FEP}}}{d_{\mathrm{TPU}}}+\varepsilon_{\mathrm{FEP}}\right)$ which, under the premise of using FEP as blocking layers, most effectively can be conducted by decreasing the thickness of the FEP film or increasing the thickness of the TPU spacer. The limit of such a reduction would be reached for $\frac{2d_{\mathrm{FEP}}}{d_{\mathrm{TPU}}}=0,\;$which results in a gain of approximately 20% compared with the present geometry.

**Figure 7:**
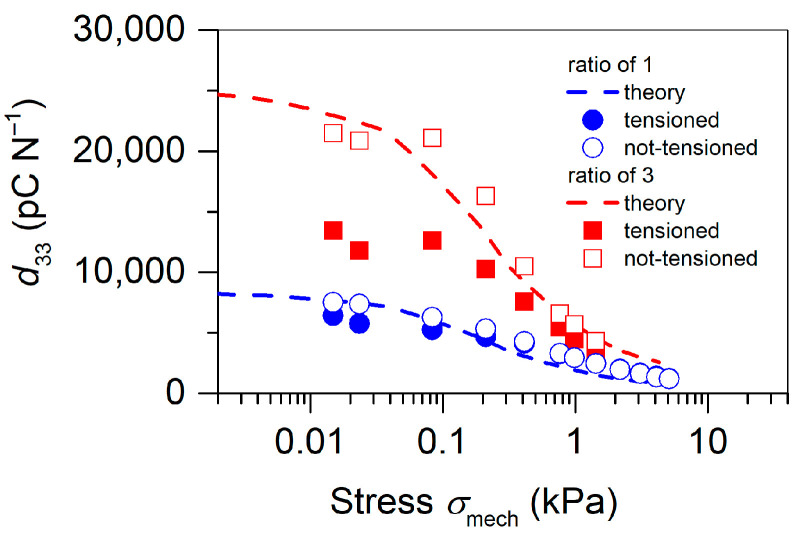
Quasi-static piezoelectric $d_{33}$ coefficients versus stress for the alternating FEP/TPU/FEP–FEP/air/FEP sandwiches with different $\frac{w_{\mathrm{air}}}{w_{\mathrm{TPU}}}\;$ ratios as indicated. The symbols represent the experimental results, while the dashed lines correspond to theoretical calculations utilizing Equation (9). The model parameters used are given in the text.

A much more effective method is the variation of the $\frac{w_{\mathrm{air}}}{w_{\mathrm{TPU}}}\;$ratio. For example, changing this ratio from 1 to 3 will, according to Equation (9), triple the $d_{33}$ coefficient and not change any of the other variables used in Equation (9). Experimentally, the ratio of 3 is easy to implement by cutting off every second TPU channel in the already printed grid with the ratio of 1. The corresponding theoretical results are shown in Figure 7 as dashed lines, while the measured $d_{33}$ coefficients at various loads are also depicted in Figure 7 by symbols for devices polarized at 25 °C. The stress dependence of $Y_{\mathrm{TPU}}$ was thereby taken from Figure 6, and the above derived breakdown field strength $E_{B}$ = 66.1 kV cm^−1^ was utilized.

Since the measurements of the $d_{33}$ coefficients were carried out at rather low stress values, it was necessary to take into account the initial tension of the samples in the radial direction introduced by the clamping in the sample holder. The influence of such radial tension on the $d_{33}$ coefficients was measured for tensioned and non-tensioned specimens. The corresponding results are also depicted in Figure 7. It can be seen that the release of the initial tension had an especially large effect on the piezoelectric response of the sandwich with the ratio $\frac{w_{\mathrm{air}}}{w_{\mathrm{TPU}}}$ = 3. This can be explained by the fact that the radial tension induced by stretching the sandwich through the embossment and groove of the sample holder compressed mainly the TPU spacer of the sandwich to a point where the induced pre-stress exceeded 0.1 kPa and the Young’s modulus started to increase as can be seen in Figure 6. After removing the initial pre-stress by loosening the screws (see Figure 2b), the sample revealed the values of the $d_{33}$ coefficients, which were in excellent agreement with the theoretical estimates.

In general, it can be stated that the here presented model provided reliable prediction of the piezoelectric response of elastomer-spaced piezoelectrets at different stress levels. The observed decrease of the $d_{33}$ coefficients under higher loads can be explained by the increase in the Young’s modulus depicted in Figure 6 and has been reported for other ferroelectrets as well [18,19,23,26,34,37]. It should also be noted that the quasi-static $d_{33}$ values at low stresses of approximately 8000 pC N^−1^ and 22,000 pC N^−1^ obtained for samples with the $\frac{w_{\mathrm{air}}}{w_{\mathrm{TPU}}}$ ratios of 1 and 3, respectively, seemed to significantly exceed those for the already published ferroelectrets and compared to the values reported for the unipolar electret microphone when considered as a piezoelectric material [11,55].

**Figure 8:**
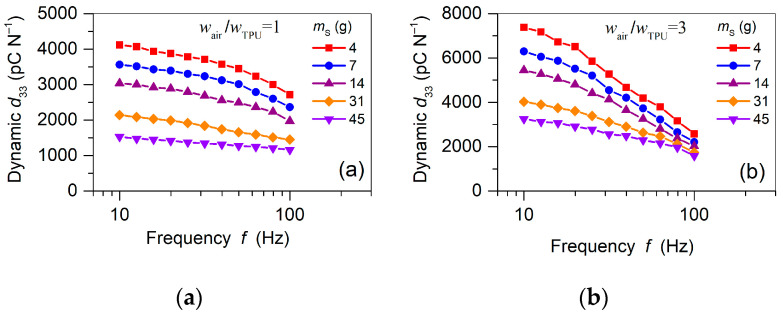
Dynamic piezoelectric $d_{33}$ coefficients versus frequency of the different seismic masses, $m_{S}$, as indicated and a constant acceleration of 3 m s^−2^: (**a**) sample with the $\frac{w_{\mathrm{air}}}{w_{\mathrm{TPU}}}$ ratio of 1; (**b**) sample with the $\frac{w_{\mathrm{air}}}{w_{\mathrm{TPU}}}$ ratio of 3. Both samples were poled at 25 °C.

In addition to the quasi-static piezoelectric $d_{33}$ coefficients, the dynamic $d_{33}$ coefficients were measured. Figure 8 shows the frequency dependencies of the piezoelectric response obtained for various seismic masses, $m_{S}$, and a constant acceleration of 3 m s^−2^ for samples with a different width ratio,$\;\frac{w_{\mathrm{air}}}{w_{\mathrm{TPU}}}$. It can be observed that the dynamic $d_{33}$ coefficients for both specimens displayed remarkable dispersion over the measured frequency window, especially for low $m_{S}.$ Increasing $m_{S}$ attenuated the piezoelectric response, which at a frequency of 10 Hz gradually decreased from approximately 7500 pC N^−1^ to 3000 pC N^−1^ for the sample with the width ratio of 3 (Figure 8b). As in the case of quasi-static $d_{33}$ coefficients, the observed decrease in the dynamic coefficients for increasing $m_{S}$ can be explained by an increase in the elastic modulus [55]. The obtained values for the dynamic $d_{33}$ coefficients were considerably smaller than their static counterparts shown in Figure 7. However, this seems to be a common property of ferroelectrets, where the dynamic piezoelectric coefficients can reach only 30–50% of the static ones, mainly caused by the difference between static and dynamic Young’s moduli [55,56].

**Figure 9:**
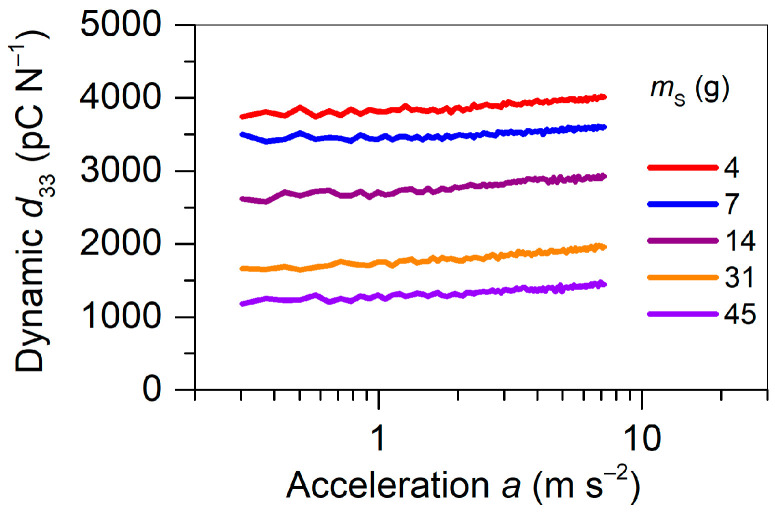
Dynamic $d_{33}$ coefficient as a function of acceleration measured at 30 Hz and different seismic masses, $m_{S}$, as indicated for the FEP/TPU/FEP–FEP/air/FEP sandwiches with the width ratio of 1 polarized at 25 °C.

The dynamic $d_{33}$ was also investigated as function of acceleration applied to the device loaded by different seismic masses, $m_{S},\;$while maintaining a constant frequency of 30 Hz. Corresponding experimental results for the same set of $m_{S}$ values as before (see Figure 8) are shown in Figure 9 for the sample with the width ratio of 1. It can be realized that the piezoelectric response exhibited a high and linear performance for accelerations up to 7.5 ${m\;s}^{-2}$, which is much improved compared to that of recently reported performances for ferroelectrets used for cantilever-based energy harvesting [57,58]. In general, the dynamic $d_{33}$ obtained for the FEP/TPU/FEP–FEP/air/FEP sandwiches significantly exceeded the typical values for classical bulk polymer ferroelectrics, such as PVDF, and even exceeded the best PZT ceramics [5]. Such results are promising for applications in highly sensitive sensors, accelerometers, and vibration energy harvesters based on the dynamic $d_{33}$ effect [10,11].

## 4. Conclusions

In this work, a new strategy for three-layer piezoelectret sandwiches was introduced. The sandwiches consisted of two functional segments both being sandwiched between layers of 25 µm thick FEP films. As an intermediate layer, a 3D-printed, 270 ± 5 µm thick, ultra-soft TPU elastomer grid with periodically placed air tunnels was used. The width of the TPU stripes was chosen to be 1 mm, and the widths of the air tunnels were 1 and 3 mm, respectively. Thus, the TPU stripes were responsible for the mechanical properties, whereas the air-filled regions were responsible for the piezoelectric properties after corona poling in an electric field exceeding the Paschen breakdown of air. The proposed design resulted in the formation of thermally stable, macroscopic electric dipoles in the FEP/air/FEP tunnels. The element FEP/TPU/FEP served as a restoring force element with an extremely low Young’s modulus under compressive load. The combination of these two elements in the form of alternating FEP/TPU/FEP–FEP/air/FEP segments guarantees a high longitudinal piezoelectric response, which for the width ratio of air tunnel to TPU stripe of 1 at small loads was on the order of 8000 pC N^−1^ for the quasi-static and approximately 4000 pC N^−^^1^ for dynamic $d_{33}$ coefficients. Using a width ratio of 3, values of up to 22,000 pC N^−1^ and 7500 pC N^−1^ were experimentally confirmed for quasi-static and dynamic coefficients, respectively. A practical advantage of the fluoropolymer–elastomer ferroelectret discussed in this paper was the linearity of the piezoelectric response for accelerations of up to 0.7× *g*, where *g* is the gravity of Earth. This distinguishes the present structure from other ferroelectrets, for which frequently published extrapolated values for 1× *g* were reported whereas the real power saturated at much smaller accelerations [11,57,58].

The work also addressed the long-term stability of the piezoelectric response. In particular, it was shown that the $d_{33}$ coefficients of samples polarized at room temperature lost approximately 40% of its initial value in 180 days in ambient laboratory conditions. TSDC measurements revealed that the origin of this loss was the well-known discharge process due to holes released from shallow traps in the FEP films [46]. A polarization of the sandwich at +120 °C greatly improved the discharge process and thereby stabilized the piezoactivity considerably.

The obtained experimental and theoretical results constitute an excellent basis for further optimization of sandwiched ferroelectrets. Especially interesting would be to optimize the geometrical effects and the electromechanical properties such as the extreme softness of the elastomer by air inclusion. Another important challenge is the ability to 3D print multilayer structures in a one-stage printing process, which would greatly simplify the manufacturing process and variability of such devices resulting in an improved overall electromechanical performance.

## Figures and Tables

**Table 1:** Printing parameters for the Varioshore TPU grid.

Extruder temperature	220 °C
Temperature of the heatbed	30 °C
Layer height	0.25 mm
Number of layers	1
Fan	disabled
Extrusion multiplier	0.70
Filament diameter	1.75 mm
Extrusion width	0.42 mm
Nozzle diameter	0.40 mm
Printing velocity	20 mm s^−1^

## Data Availability

Data are contained within the article.
